# Supplementary material for: EjFAD8 Enhances the Low-Temperature Tolerance of Loquat by Desaturation of Sulfoquinovosyl Diacylglycerol (SQDG)
Source: Int J Mol Sci. 2023 Apr 8;24(8):6946. doi: 10.3390/ijms24086946 (PMC10138649; doi:10.3390/ijms24086946)
Supplement: Supplementary file 1 [file ijms-24-06946-s001.zip › ijms-2265615-supplementary.pdf]

Supplementary material

EjFAD8 Enhances the Low-Temperature Tolerance of Loquat by Desaturation of Sulfoquinovosyl Diacylglycerol (SQDG)

Xun Xu [1,2], Hao Yang [1,2], Xiaodong Suo [1,2], Mingxiu Liu [1,2], Danlong Jing [1,2], Yin Zhang [1,2], Jiangbo Dang [1,2], Di Wu [1,2], Qiao He [1,2], Yan Xia [1,2], Shuming Wang [1,2], Guolu Liang [1,2] and Qigao Guo [1,2,*]

[1] Key Laboratory of Horticulture Science for Southern Mountains Regions of Ministry of Education, College of Horticulture and Landscape Architecture, Southwest University, Chongqing 400715, China; xuxun8433@163.com (X.X.); xiatian87285@163.com (H.Y.); sxd18883770582@163.com (X.S.); lmx2004121733@163.com (M.L.); jingdanlong@swu.edu.cn (D.J.); zhangyin2021@swu.edu.cn (Y.Z.); jiangbodang@swu.edu.cn (J.D.); wudisuper@swu.edu.cn (D.W.); heqiao2005@126.com (Q.H.); yansummer@swu.edu.cn (Y.X.); wangsm2018@swu.edu (S.W.); lianggl@swu.edu.cn (G.L.)

[2] Academy of Agricultural Sciences of Southwest University, State Cultivation Base of Crop Stress Biology for Southern Mountainous Land of Southwest University, Chongqing 400715, China

* Correspondence: qgguo@126.com; Tel.: +86-023-68250383

Supplementary Figures and Tables

1.1 Supplementary Figures

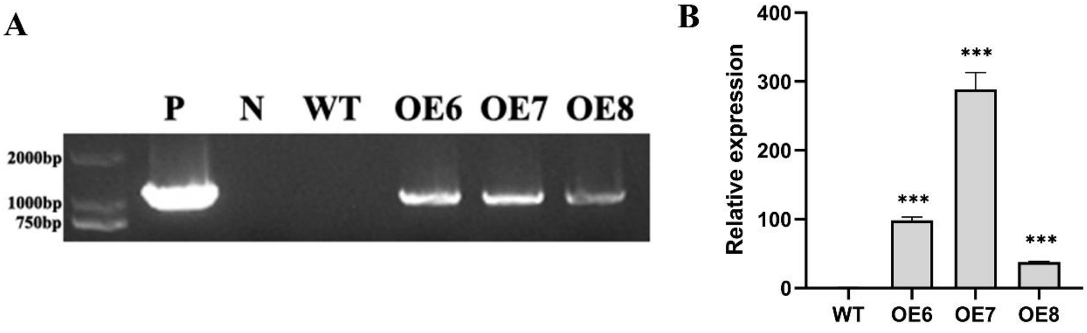

Supplementary Figure 1. (**A**) Electrophoretic results of semi-quantitative PCR for EjFAD8 in Arabidopsis thaliana. P, positive; N, negative. (B) The identification of overexpression *EjFAD8* transgenic *Arabidopsis*.

1.2 Supplementary Tables

Supplementary Table S1 | Primers used for gene cloning and vector construction

Primer name	Primer F sequences（5’-3’）	Primer R sequences（5’-3’）
EjFAD8	ATGGCGACTTGGGTCCTCTC	CTAGGTTGAAGTGGTACTCAC
pCAMBIA2300-EjFAD8	gacagggtacccggggatccATGGCGACTTGGGTCCTCTC	gtgtcgactctagaggatccGGTTGAAGTGGTACTCACTGC

Supplementary Table S2 | Gene source and ID

Gene	Specie	ID
AtFAD7	*Arabidopsis thaliana*	NP_187727.1
AtFAD8	*Arabidopsis thaliana*	NP_001190230.1
CiFAD8	*Carya illinoinensis*	XP_042942544.1
CsFAD8	*Cannabis sativa*	XP_030488828.1
MdFAD7	*Malus domestica*	XP_028945489.1
MdFAD8	*Malus domestica*	NP_001280916.1
PaFAD7	*Prunus avium*	XP_021830308.1
PaFAD8	*Prunus avium*	XP_021805633.1
PbFAD8	*Pyrus bretschneideri*	XP_048420918.1
PdFAD7	*Prunus dulcis*	XP_034221835.1
PdFAD8	*Prunus dulcis*	XP_034202239.1
RcFAD7	*Rosa chinensis*	XP_024189650.1
RcFAD8	*Rosa chinensis*	XP_024177352.1
ToFAD7	*Trema orientale*	PON92222.1
ToFAD8	*Trema orientale*	PON86591.1

Supplementary Table S3 | Primers used for quantitative real-time PCR (qRT-PCR)

Primer name	Primer F sequences（5’-3’）	Primer R sequences（5’-3’）
EjFAD8	CAGCGAACTGTTTCTCCCAA	CTTGGACAGGTCCCATTGTG
AtActin	TATCGCTGACCGTATGAG	CTGAGGGAAGCAAGAATG
EjActin	ATCCTTCGTCTGGACCTTGC	GACAATTTCCCGTTCAGCAGT
AtCBF1	AATGTTTGGCTCCGATTACG	CCCACTTACCGGAGTTTCTT
AtCBF2	GACCTTGGTGGAGGCTATTT	ATCCCTTCGGCCATGTTATC
AtCBF3	TTATATGCACGATGAGGCGA	ATGATTCCACTGTACGGACG
AtICE1	GTTCGGGAATGAGGAGGTTT	AACACTCTCAGCCGCTTTAC
AtICE2	TCCACAAACGCTGTCTTACC	GTTCACTGCCTTTCCTTCTCT
AtRD29A	CTTGTCGACGAGAAGCAAAGAA	TCTTGATGGAGAATTCGTGTCC
AtCOR47	TGTCATCGAAAAGCTTCACCGA	ACCGGGATGGTAGTGGAAACTG
AtKIN1	ATGCCTTCCAAGCCGGTCAGAC	CCGGTCTTGTCCTTCACGAAGT
AtCOR15A	GTCGTCGTTTCTCAACGCAAGA	GCTTTCTCAGCTTCTTTACCCA
AtFAD4	CCAACATCCCAAAAAGCCAC	GAGGAAGATTCACAGTGCGT
AtFAD6	TTGGTGGAAGACATTGTGGG	AACATGTTGGTTTTGGCGTG
AtFAD7	CTCTATTGGCTCGCTCAAGG	CCGACCACACTGTTCAACTT
AtFAD8	ATCCGAGGAAGACACGGAGA	GGCTGCTCTTATATCCGCCA

AtMGD1	TCAAATCGTTCATGTGGTTCTC	GCCCTTTTTGCCACTTCTGT
AtMGD2	TGTGAGGCCATCTTTTGCAC	TTCCCCTCCTCCCATCAGTA
AtMGD3	TCATCACTAAGGCTGGTCCG	CGTTGCCTTTTTCCTGTCCA
AtDGD1	TGTGTGCAGATCACCCTTCA	AGTGGCTCTTTCGTCATTGC
AtDGD2	CACCAAAAGGAACTTGCCGA	CGGGCTGCTTCTTTGATCTC
AtSQD1	TAAGCCGTTTGTTTCAGGGC	ACGCAGCTTTTTCTCGGTTT
AtSQD2	ACTTGGCCTTGTGGTTCTTG	TCCGGTTTTTCCCTCCTGAT
AtPGP1	CGCAGATGACAGCACTAACC	ACATAAAGCAAGCCAGCACC
